# Supplementary material for: Integrative genomics identifies a convergent molecular subtype that links epigenomic with transcriptomic differences in autism
Source: Nat Commun. 2020 Sep 25;11:4873. doi: 10.1038/s41467-020-18526-1 (PMC7519165; doi:10.1038/s41467-020-18526-1)
Supplement: Supplementary file 3 — Description of Additional Supplementary Information [file 41467_2020_18526_MOESM3_ESM.pdf]

Description of Additional Supplementary Files

File Name: Supplementary Data 1
Description: ASD and Control sample metadata.

File Name: Supplementary Data 2
Description: Differential mRNA expression.

File Name: Supplementary Data 3
Description: Differential miRNA expression and target prediction.

File Name: Supplementary Data 4
Description: Differential DNA methylation and comethylation networks.

File Name: Supplementary Data 5
Description: Differential H3K27ac and gene links.
