# Supplementary material for: Integrative genomics identifies a convergent molecular subtype that links epigenomic with transcriptomic differences in autism
Source: Nat Commun. 2020 Sep 25;11:4873. doi: 10.1038/s41467-020-18526-1 (PMC7519165; doi:10.1038/s41467-020-18526-1)
Supplement: Supplementary file 9 — Reporting summary [file 41467_2020_18526_MOESM9_ESM.pdf]

nature research

Corresponding author(s): Daniel Geschwind

Last updated by author(s): Jul 9, 2020

Reporting Summary

Nature Research wishes to improve the reproducibility of the work that we publish. This form provides structure for consistency and transparency in reporting. For further information on Nature Research policies, see Authors & Referees and the Editorial Policy Checklist .

Statistics

For all statistical analyses, confirm that the following items are present in the figure legend, table legend, main text, or Methods section.

n/a	Confirmed	
☐	☒	The exact sample size (n) for each experimental group/condition, given as a discrete number and unit of measurement
☒	☐	A statement on whether measurements were taken from distinct samples or whether the same sample was measured repeatedly
☐	☒	The statistical test(s) used AND whether they are one- or two-sided<br>*Only common tests should be described solely by name; describe more complex techniques in the Methods section.*
☐	☒	A description of all covariates tested
☐	☒	A description of any assumptions or corrections, such as tests of normality and adjustment for multiple comparisons
☐	☒	A full description of the statistical parameters including central tendency (e.g. means) or other basic estimates (e.g. regression coefficient) AND variation (e.g. standard deviation) or associated estimates of uncertainty (e.g. confidence intervals)
☐	☒	For null hypothesis testing, the test statistic (e.g. F, t, r) with confidence intervals, effect sizes, degrees of freedom and P value noted<br>*Give P values as exact values whenever suitable.*
☒	☐	For Bayesian analysis, information on the choice of priors and Markov chain Monte Carlo settings
☒	☐	For hierarchical and complex designs, identification of the appropriate level for tests and full reporting of outcomes
☐	☒	Estimates of effect sizes (e.g. Cohen's d, Pearson's r), indicating how they were calculated

Our web collection on statistics for biologists contains articles on many of the points above.

Software and code

Policy information about availability of computer code

Data collection	No software was used for data collection, all data was downloaded from public repositories.
Data analysis	Data analysis scripts are available on github: https://github.com/dhglab/ASD-Integration-Subtypes-Manuscript . Softwares: CQN R package version 1.16, Combat from SVA R package version 3.18, BWA-MEM version 0.7.17, Picard Tools version 2.13.2, SNFtool R package version 2.2.1, R version 2.3.2, WGCNA R package version 1.51, nlme R package version 3.1-125, TargetScan version 7.2, GREAT version 4.0.4, g:Profiler R package version 0.7, LD-score regression version 1.0.0

For manuscripts utilizing custom algorithms or software that are central to the research but not yet described in published literature, software must be made available to editors/reviewers. We strongly encourage code deposition in a community repository (e.g. GitHub). See the Nature Research guidelines for submitting code & software for further information.

Data

Policy information about availability of data

All manuscripts must include a data availability statement. This statement should provide the following information, where applicable:
- Accession codes, unique identifiers, or web links for publicly available datasets
- A list of figures that have associated raw data
- A description of any restrictions on data availability

Full range of values underlying heatmaps in Figures 2f, 2i, 4e, 4f, 5a, 5b and Supplementary Figures 7e, 7g, 8e, 8f, 9f, 9g, 10f, 10g, 11a, 11b, 11c, 11d, 13a, 13b, and 13c are provided as source data. Harvard Autism Tissue Program [https://hbtrc.mclean.harvard.edu/], NIH Neuro Brain Bank [http://www.medschool.umaryland.edu/btbank/], Oxford Brain Bank [https://www.hra.nhs.uk/planning-and-improving-research/application-summaries/research-summaries/the-oxford-brain-bank/], MRC London Brain Bank [https://brainbanknetwork.cse.bris.ac.uk/], Raw data for mRNA, miRNA, DNA methylation, and H3K27ac from ASD and control brains (Synapse.org accession number syn4587609), Gencode [https://www.gencodegenes.org/], hg19 genome [http://genome.ucsc.edu/], TargetScan DB [http://www.targetscan.org/cgi-bin/targetscan/data_download.vert72.cgi], Psychencode eQTL and Hi-C datasets [http://resource.psychencode.org/] (Synapse.org accession number: syn10248174 for NeuN-, syn10248215 for NeuN+), LD-score regression model [https://github.com/

bulik/ldsc/wiki/Partitioned-Heritability], Gene expression of ASD and control brains from other cortical regions (Synapse.org accession number syn11242290).

Field-specific reporting

Please select the one below that is the best fit for your research. If you are not sure, read the appropriate sections before making your selection.

- [x] Life sciences
- [ ] Behavioural & social sciences
- [ ] Ecological, evolutionary & environmental sciences

For a reference copy of the document with all sections, see nature.com/documents/nr-reporting-summary-flat.pdf

Life sciences study design

All studies must disclose on these points even when the disclosure is negative.

Sample size	No sample size determinations were made in this study, all datasets were previously published and publicly available. We used all of the samples available from the published studies as our starting dataset.
Data exclusions	To balance ASD and control cohorts with respect to donor age, we removed donors with age < 10 years in the miRNA and DNA methylation datasets. To balance the 4 datasets with respect to brain bank, we excluded samples from the Oxford and MRC London Brain Banks in the DNA methylation and Histone Acetylation datasets. We also did not analyze any samples originating from the cerebellum from the previous publications.
Replication	No replication dataset was generated. We compared our results to those from previous ASD publications (Parikshak et al. 2016, Wu et al. 2016, Sun et al. 2016, Wong et al 2019) to ensure replication.
Randomization	No randomization was performed. We included biological and technical covariates in differential analyses. For mRNA expression, we ran differential expression analysis by fitting a linear mixed effect model for each gene: Expression ~ Diagnosis + Age + Sex + Region + RIN + Brain bank + Sequencing batch + seqStatPC1 + seqStatPC2 + seqStatPC3 + seqStatPC4 + seqStatPC5 as fixed effects, and brainID as a random effect. For miRNA expression, we ran differential expression analysis by fitting a linear mixed effect model for each miRNA transcript: Expression ~ Diagnosis + Age + Sex + Region + RIN + Brain bank + Proportion of reads mapping to exons + log10(Sequencing depth) + PMI as fixed effects, and brainID as a random effect. For DNA methylation, we ran differential methylation analysis by fitting a linear mixed effect model for each promoter and each gene body: Methylation ~ Diagnosis + Age + Sex + Region + Brain bank + Batch + CET as fixed effects, and brainID as a random effect. For histone acetylation, we ran differential acetylation analysis by fitting a linear mixed effect model for each H3K27ac peak: Acetylation ~ Diagnosis + Age + Sex + Region + Brain bank + CET + Fraction of reads in peaks + Duplicate read fraction + Aligned read fraction as fixed effects, and brainID as a random effect.
Blinding	No blinding was performed. All of the datasets were previously published and we had access to the metadata with diagnostic status for each individual.

Reporting for specific materials, systems and methods

We require information from authors about some types of materials, experimental systems and methods used in many studies. Here, indicate whether each material, system or method listed is relevant to your study. If you are not sure if a list item applies to your research, read the appropriate section before selecting a response.

Materials & experimental systems

n/a	Involved in the study	
[x]	[ ]	Antibodies
[x]	[ ]	Eukaryotic cell lines
[x]	[ ]	Palaeontology
[x]	[ ]	Animals and other organisms
[x]	[ ]	Human research participants
[x]	[ ]	Clinical data

Methods

n/a	Involved in the study	
[x]	[ ]	ChIP-seq
[x]	[ ]	Flow cytometry
[x]	[ ]	MRI-based neuroimaging
